# Myths in the laboratory diagnosis of HIV infection

**DOI:** 10.1080/22221751.2019.1656550

**Published:** 2019-08-26

**Authors:** Hongzhou Lu, Yi-Wei Tang

**Affiliations:** a Department of Infectious Diseases, Shanghai Public Health Clinical Center, Shanghai, People’s Republic of China; b Department of Infectious Diseases, Huashan Hospital, Fudan University, Shanghai, People’s Republic of China; c Department of Laboratory Medicine, Department of Internal Medicine, Memorial Sloan Kettering Cancer Center, New York, USA; d Department of Pathology and Laboratory Medicine, Weill Medical College of Cornell University, New York, USA

Significant technical advances have been made in the detection and characterization of human immunodeficiency virus (HIV) since this virus was determined to be the pathogen causing AIDS [1,2]. For the laboratory diagnosis of HIV infections, laboratorians/clinical microbiologists first determine whether a patient is infected and, if an HIV infection diagnosis is established, they monitor antiretroviral therapy. Theoretically, an HIV infection can be diagnosed and monitored by any of five possible ways: (i) direct microscopic examination such as visualization of an HIV virion by electronic microscopy, (ii) cultivation and identification of HIV by suspension lymphocyte culture, (iii), detection of HIV viral antigens, (iv) measurement of HIV-specific immune responses, and (v) detection and quantification of HIV-specific nucleic acids [2,3]. Practically speaking, the diagnosis and monitoring of HIV infection are done by serologic and molecular methods. While serology tests covering both HIV-specific antigens and antibodies remain the mainstay for the diagnosis of HIV infections, molecular assays are being used increasingly for antiretroviral monitoring and drug resistance determination.

On 25 November 2018 during the second World Summit of Human Gene Editing meeting, Jiankui He, a biophysicist from the Southern Univeristy of Science and Technology in Shenzhen, China, presented the gene-editing project that led to the birth of two baby girls with man-made C–C chemokine receptor type 5 (CCR5) mutations [4–6]. This generated misleading information in the field of prevention and treatment of HIV infections. We feel it is necessary to explore several similar “myths” in the field of clinical virology in relation to the laboratory diagnosis of HIV infections.

## Myth 1: Nucleic acid amplification test is the test of choice for diagnosis

Molecular methods amplify and detect HIV-1 specific nucleic acids in vitro by PCR and other in vitro enzyme-mediated nucleic acid amplification (NAA) techniques. Serology methods, on the other hands, detect and identify HIV-1-specific antibodies and antigens in serum by classical and rapid immunoassays [3]. Although NAA tests play important roles in monitoring HIV infections by providing HIV viral loads and determining antiviral resistance, serology remains the primary test of choice for the diagnosis of HIV infections. Fourth-generation “combo” immunoassays for both HIV antibodies and the P24 antigen are rapid and sensitive [7,8]. Only in certain unusual events and specific populations is NAAT used for diagnosis, such as HIV infections in neonates and transplant recipients.

Misuse of NAAT in the diagnosis of HIV infection can lead to false positive results. False-positive HIV-1 NAAT tests have been observed following treatment with therapies that use lentiviral vectors [9,10]. Laetsch et al. described four patients with positive Roche Cobas HIV results following tisagenlecleucel or CTL119 infusion. All patients were HIV negative at screening prior to chimeric antigen receptor (CAR) T-cell therapy, and negative results were subsequently confirmed using HIV-1 NAAT or antigen tests by another commercial vendor. No patient developed replication-competent lentivirus, as evaluated by vesicular stomatitis virus glycoprotein quantitative PCR; thus, these cases represent false-positive HIV-1 NAT test results [11]. We recently described three unique case scenarios in which CAR-T cell immunotherapy interfered with HIV molecular testing [12]. When NAAT tests are used for diagnosis of HIV infections, positive HIV RNA screening results warrant critical interpretation when the patient has a low risk of the disease. In addition, treatment with lentiviral-derived CAR T-cells may lead to false-positive results on HIV RNA testing.

## Myth 2: The rapid HIV antibody test is the test of choice for the early diagnosis of HIV infection

The logistics of classical HIV immunoassays require phlebotomy, and, typically, a follow-up visit fo test results after the specimen has been processed. In contrast, rapid HIV immunoassays are single-use devices that use either flow-through or lateral flow platform which allows the use of direct and unprocessed specimens in point of care [1,3]. HIV rapid tests became available to fulfil the need to promptly determine the serostatus of persons prior to surgical operations, organ transplantation, and maternal labor/delivery.

Rapid testing, in contrast, is usually completed in about 20–30 min, thus making them ideal for testing and counselling in primary health care sites and mobile clinics [1]. In addition, rapid HIV self-test kits have the potential to increase testing rates around the globe, and thereby lead to reductions in HIV morbidity and mortality [13]. One of the rapid tests, OraQuick, uses a painless oral fluid collection technique that is preferred by many over finger-stick whole blood methods [13]. A recent literature review found that among HIV self-testing kits, consumers would accept the test if available at a relatively low cost, and preferred the oral-based HIV self-testing method over the blood-based test [14]. However, the “rapid” means that the test can be done within a short time and has nothing to do with an ability to reach an earlier diagnosis. As a matter of fact, most rapid antibody tests, (e.g. OralQuick) are less sensitive and less specific than the routinely used immunoassays – especially fourth generation antibody–antigen combo tests [1,15]. For early and accurate diagnosis, any rapid test results should be confirmed by a fourth-generation immunoassay following the WHO- or CDC-recommended testing algorithm.

## Myth 3: No tropism testing is needed as HIV uses CCR5 solely as coreceptor for early entry

HIV replication begins with the attachment of the virus to the target cell via the interaction of viral envelope glycoproteins (gp120) and host cellular receptor CD4. This binding results in gp120 conformational changes that allow the virus to interact with a cellular coreceptor which determines cell tropism. Several coreceptors have been revealed including CCR5, CCR3 and CXCR4. While the interaction with CXCR4 occurs primarily with T-cell-tropic, syncytium-inducing viruses, the CCR5 and CCR3 are involved in attachment of macrophage-tropic, non-syncytium-inducing HIV [16,17]. An entry inhibitor, maraviroc, has led to a need for viral tropism assays because the drug is only effective against viruses that use the CCR5 as a coreceptor for entry.

Tropism assays such as the Trofile DNA [18] or the SensiTrop II test [19] are commercially available to identify candidate patients infected with exclusively R5 HIV as the viral population that uses CXCR4 would unlikely be affected by the CCR5-targeting therapy. An international HIV-1 coreceptor proficiency panel test results demonstrated that genotypic tropism prediction is a safe procedure for clinical purposes [20]. The tropism assay must be performed prior to initiating maraviroc to determine whether the virus is CCR5-tropic. Tropism can be determined either phenotypically or genotypically. Depending on which cells they infect, the viruses are then designated CXCR4-, CCR5-, or dual/mixed-tropic in the phenotypic Trofile assay (145). In contrast, the genotypic SensiTrop assay uses a heteroduplex tracking assay combined with sequence analysis to identify minor viral populations that may be CXCR4-tropic. Resistance to maraviroc has been reported, as have data showing the development of mutations that allow the virus to use CXCR4 coreceptors, or mutations that lead to structural changes in the envelop that prevent the drug from being effective [19,21].

## Conclusions

Advanced diagnostic techniques have been quickly developed and implemented as the mainstay for laboratory diagnosis and monitoring of HIV-1 infections. Serology tests using fourth-generation “combo” immunoassays for both HIV antibodies and antigens remains the primary test of choice for diagnosis of HIV infections. Inappropriate use of NAAT may result in false positives, especially in patients receiving immunotherapy. HIV rapid antibody tests provide results quickly in primary health care settings and mobile clinics, but they are usually not as sensitive and specific as antibody–antigen combo immunoassays. HIV-1 entry into macrophages and T helper cells is mediated not only through the interaction of gp120 with the CD4 molecule on the target cells, but also with its chemokine coreceptors. HIV tropism, which refers to the cell type that the HIV infects and replicates in, can be determined phenotypically or genotypically. However, the use of a coreceptor alone does not explain viral tropism, as not all CCR5-tropic viruses are able to use CCR5 on macrophages for a productive infection. Thus, the report on the editing of the CCR5 genes of the twin embryos to “prevent them from contracting HIV” is misleading and perhaps wishful thinking at best. Correct understanding of these myths in the laboratory diagnosis of HIV infections is important to provide rapid and evidence-based therapeutic action.
